# Aeromonas hydrophilia Infection in an Immunocompromised Host

**DOI:** 10.7759/cureus.20834

**Published:** 2021-12-30

**Authors:** Yi Zhao, Julie Alexander

**Affiliations:** 1 Internal Medicine, Methodist Health System, Dallas, USA; 2 Infectious Diseases, Methodist Health System, Dallas, USA

**Keywords:** aeromonas hydrophila, immunocompromised, osteomyelitis, soft tissue infection, hiv

## Abstract

*Aeromonas hydrophila* is an anaerobic, gram-negative rod-shaped bacterium that is commonly found in aquatic environments. This organism can cause a wide range of infections in humans, including gastroenteritis, septicemia, and skin and soft tissue infections. We herein describe a case of bacteremia due to *Aeromonas hydrophila* secondary to a soft tissue infection of the fingers that resulted in osteomyelitis in an immunocompromised patient.

## Introduction

The *Aeromonas* species are flagellated, facultative, anaerobic, non-spore-forming, catalase-negative, and oxidase-positive Gram-negative rods in the family Aeromonadaceae [1]. Four *Aeromonas* serotypes are associated with more than 95% of various infections in humans: *A. caviae*, *A. dhakensis*, *A. veronii*, and *A. hydrophila*, in order of prevalence [2]. *A. hydrophila* is found in soil and a variety of freshwater and seawater aquatic environments. It was one of the more prevalent species identified in aquatic injuries after the 2001 Thailand tsunami [3]. *Aeromonas* spp. have been historically recognized as a cause of gastroenteritis, but can also cause septicemia, wound, and soft tissue infections. Cellulitis is the most common presentation, although myonecrosis, gangrene, and osteomyelitis have also been reported [1]. We present a case of bacteremia due to *A. hydrophila* secondary to a soft tissue infection of the fingers that resulted in osteomyelitis in an immunocompromised patient.

## Case presentation

**Figure 1:**
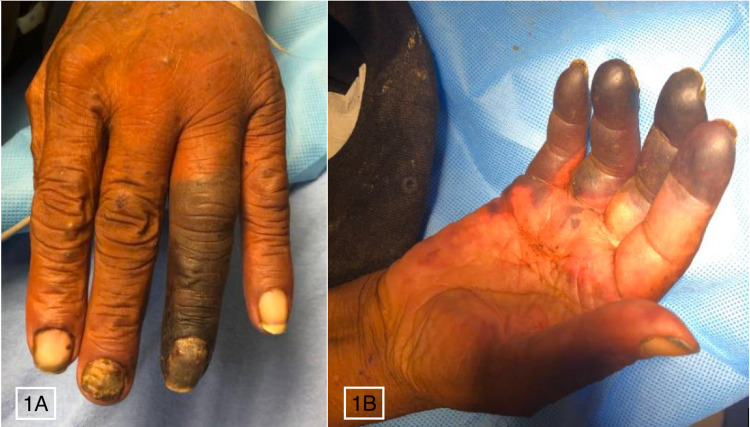
Dry gangrene of the left fourth and fifth digits (A) and the right second, third, fourth, and fifth digits (B).

A homeless 62-year-old male was bought to the emergency department (ED) after being found in a field. He had a past medical history significant for chronic alcoholism and human immunodeficiency virus with a CD4+ T cell count of 138 cells/mm^3 and a viral load of 138,827 copies/mL, but did not take antiretroviral medications. He was alert and orientated on arrival but unaware of the details leading up to this hospitalization. On physical examination, he had dry gangrene of the left fourth and fifth digits and right second, third, fourth, and fifth digits without any open wounds (Figure 1). Of note, he was seen in the ED three days prior for acute alcohol intoxication and diffuse myalgia, without documented necrotic digits or skin changes. He denied any recent trauma or insect bites to his hands but complained of finger pain for a couple of days before the current presentation. A computed tomography (CT) scan of both hands showed soft tissue swelling and a small amount of soft tissue gas in the fourth digits of both hands. A hepatic workup was conducted due to his elevated liver enzymes and total bilirubin level on admission combined with his history of alcohol abuse. An acute hepatitis panel was unremarkable; further workup with a liver ultrasound revealed hepatomegaly and steatosis, but no evidence of cirrhosis. 

He was admitted for dry gangrene of multiple fingers. He met two out of the four systemic inflammatory response syndrome criteria with an elevated white blood cell count (15.4 x 103/µL), tachycardia (115 beats per minute), and an elevated lactic acid level (5.88 mmol/L). He was started on broad-spectrum antibiotics, vancomycin, cefepime, and clindamycin. A hand surgeon was consulted, and after reviewing the bilateral hand CT, they proposed that the patient was exhibiting a small amount of soft tissue gas related to defective nails instead of true gas gangrene. The surgeon’s recommendations included continuing the antibiotics and to proceed with digit amputation once a complete demarcation of gangrene was achieved. Blood cultures drawn on admission grew *A. hydrophila* in two out of two sets. Thus, antibiotics were narrowed to ceftriaxone 2g intravenously (IV) once daily and doxycycline 100mg orally twice daily per Infectious Disease Society of America recommendations [4]. The patient later complained of non-specific back pain; however, CT scans of the cervical, thoracic, and lumbar spine were unremarkable. Magnetic resonance imaging of the brain revealed multiple foci of acute or subacute infarcts in the right frontal lobe, bilateral thalami, and left occipital lobe, which was suspicious for an embolic process given the distribution. Both a transthoracic echocardiogram and a transesophageal echocardiogram showed no vegetations. On day five of admission, he eventually underwent amputation of multiple digits, including the right first, second, third, and fourth fingers, and the left fourth and fifth fingers; intra-operative tissue cultures grew *Enterococcus faecalis* and *A. hydrophila*. Pathology reports showed gangrenous necrosis with a clean margin and no evidence of malignancy. Antimicrobials were then switched to piperacillin-tazobactam for coverage of both pathogens. He completed 10 days of IV piperacillin-tazobactam post-surgery and then was switched to amoxicillin-clavulanate for 14 days for skin and soft tissue infection. He was also started on trimethoprim-sulfamethoxazole for *Pneumocystis jirovecii *prophylaxis. Incidentally, he had a negative rapid plasma reagin test, but was positive for *Treponema pallidum* antibodies. He did not have any record of prior syphilis treatment at the county health department, so he was also started on treatment for late latent syphilis of unknown duration with penicillin G benzathine 2.4 million units intramuscularly once weekly for three doses. On hospital follow-up at one week and one month, his hand wounds were healing well without further signs of infection.

## Discussion

The oral-fecal route is the most common route of entry for *Aeromonas*, followed by open wounds in the extremities [5]. A retrospective study conducted in Taiwan, an endemic area for *Aeromonas* species [6], found that 78% of 129 cases of skin and soft tissue infections attributed to *Aeromonas* had prior trauma and 30% of cases had some water exposure. *Aeromonas* infections occur more frequently during warmer months. This observation is consistent with our patient's presentation. On average, Dallas weather ranges from 64˚F to 89˚F in September, with an average precipitation of 3.33 inches [7]. Although infrequently seen, *Aeromonas* infections due to soil contaminants have been reported, including 26 mud football players who were diagnosed with *Aeromonas* infection [8]. In our patient, it was difficult to delineate the exact route of entry as he could not provide precise details leading up to the admission. However, since the patient was found in a field with recent rainfall, his soft tissue infection was suspected to be due to soil contaminants.

*Aeromonas* can cause a wide range of infections in humans, including bacteremia, pneumonia, gastroenteritis, skin and soft tissue infections, hepatobiliary infections, and necrotizing fasciitis. Although *Aeromonas* can infect healthy and immunocompromised individuals, it is more frequently isolated in patients with underlying malignancies and liver cirrhosis [1, 2]. An article published in 1995 examined 59 *Aeromonas* bacteremia cases in southern Taiwan, where researchers reported a crude fatality rate of 36% with cases predominantly clustered in the warmer months. They also found that cases predominantly affected male patients and those with underlying chronic liver diseases or neoplasms [9]. Similar results were seen in an updated study conducted in Taiwan from 2009 to 2013: the fatality rate of *Aeromonas* bacteremia patients was 41% in the cirrhosis group and 36% in the neoplasm group [10]. Skin and soft tissue infection was the third most common source of secondary infection after peritonitis and biliary tract infection. In-hospital mortality was significantly associated with underlying cancer and shock on presentation [10]. Our patient did not have evidence of cirrhosis, but was immunocompromised due to uncontrolled HIV, making him more susceptible to this pathogen.

*Aeromonas* isolates encountered in human infections are susceptible to fluoroquinolones, tetracyclines, aminoglycosides, carbapenems, monobactams, and third- and fourth-generation cephalosporins [5]. For extraintestinal infections and septicemia, there are no clinical trial data to guide the duration of therapy; therefore, treatment was guided by clinical response [1]. The patient was initially treated with ceftriaxone for *Aeromonas* bacteremia, but the antibiotics were later broadened to piperacillin-tazobactam to cover *Enterococcus faecalis *coinfection. He underwent a total of 15 days of IV antibiotics (10 days since the day of surgery), followed by 14 days of oral amoxicillin-clavulanate with no evidence of infection at his one-month outpatient follow-up.

## Conclusions

*Aeromonas hydrophila* is historically recognized as a cause of gastroenteritis, but can also cause septicemia as well as wound and soft tissue infections. *A. hydrophilia* infections should be considered in patients with soil and aquatic exposure, particularly in immunocompromised hosts. Early recognition and treatment can decrease *A. hydrophilia*-associated mortality. 
